# Exposure to Zinc Oxide Nanoparticles Increases Estradiol Levels and Induces an Antioxidant Response in Antral Ovarian Follicles In Vitro

**DOI:** 10.3390/toxics11070602

**Published:** 2023-07-12

**Authors:** Ramsés Santacruz-Márquez, Jodi A. Flaws, Luz del Carmen Sánchez-Peña, Isabel Hernández-Ochoa

**Affiliations:** 1 Centro de Investigación y de Estudios Avanzados del Instituto Politécnico Nacional, Departamento de Toxicología, Av. Instituto Politécnico Nacional 2508, Col. San Pedro Zacatenco, Mexico City 07360, Mexico; 2 Department of Comparative Biosciences, University of Illinois Urbana-Champaign, Urbana, IL 61802, USA

**Keywords:** nanoparticles, ZnO nanoparticles, antral follicles, ovary, toxicity, hormones, endocrine disruptors

## Abstract

The use of zinc oxide nanoparticles (ZnO NP) in consumer products is increasing, raising concern about their potential toxicity to human health. Nanoparticles have endocrine disrupting effects and can induce oxidative stress, leading to biomolecule oxidation and cell dysfunction. The ovary is one of the most important endocrine organs in female reproduction. Nanoparticles accumulate in the ovary, but it is unknown whether and how exposure to these materials disrupts antral follicle functions. Thus, this study tested the hypothesis that the in vitro exposure to ZnO NPs affects the steroidogenic pathway and induces oxidative stress in ovarian antral follicles. Antral follicles from CD-1 mice were cultured with ZnO NPs (5, 10, and 15 µg/mL) for 96 h. ZnO NP exposure did not affect apoptosis and cell cycle regulators at any of the tested concentrations. ZnO NP exposure at low levels (5 µg/mL) increased aromatase levels, leading to increased estradiol levels and decreased estrogen receptor alpha (*Esr1*) expression. ZnO NP exposure at 15 µg/mL induced an antioxidant response in the antral follicles as evidenced by changes in expression of antioxidant molecules (*Nrf2*, *Cat*, *Sod1*, *Gsr*, *Gpx*) and decreased levels of reactive oxygen species. Interestingly, ZnO NPs dissolve up to 50% in media and are internalized in cells as soon as 1 h after culture. In conclusion, ZnO NPs are internalized in antral follicles, leading to increased estrogen production and an antioxidant response.

## 1. Introduction

ZnO nanoparticles (ZnO NPs) are widely produced materials with many uses in different industries due to specific electronic, magnetic, and optical characteristics compared to bulk materials. Some of the most relevant and interesting characteristics of ZnO NPs include broad-spectrum UV protection, antibacterial, antifungal, anti-inflammatory, and skin healing properties [1,2]. Due to their specific properties, ZnO NPs are used in optics, electronics, and biomedical and materials sciences [3], and are very common in numerous products including adhesives, batteries, ceramics, cosmetics, glass, lubricants, paints, pigments, and supplementary foods [4,5].

The global ZnO NP market had an estimated value of $376.6 million US dollars in 2022, an amount expected to double in a ten-year period [6]. The increasing use of ZnO NPs poses an increased risk of human exposure through occupational and environmental sources. NPs are becoming known as endocrine disrupting chemicals, which means they can interfere with hormone functions, affecting several systems, including the reproductive, brain, and immune systems [7,8]. On the other hand, NP exposure has been shown to induce oxidative stress by inducing the formation of reactive oxygen species (ROS) or by affecting the antioxidant system capacity in non-reproductive tissues [9,10] and reproductive tissues [11]. The widespread use and increasing uses of ZnO NPs have raised concerns about their potential toxicity to human health [12]. Thus, studies on NP toxicity and endocrine disrupting effects are needed.

The ovary is an important endocrine organ in female reproduction because it is the site where folliculogenesis, oogenesis, and sex steroid hormone synthesis occur in a concerted manner. In coupled processes, primordial follicles, consisting of an oocyte surrounded by granulosa cells, develop into primary, secondary, antral, and ovulatory follicles through the folliculogenesis process [13]. Meanwhile, oocytes achieve maturation and the capacity to be fertilized through the process of oogenesis [14]. The antral follicles are characterized by possessing granulosa cells, two or more layers of theca cells, and a fluid-filled space named the antrum. The antral follicle is capable of synthesizing and secreting sex steroid hormones such as androgens and estrogens through the two-cell and two-gonadotropin system. Luteinizing hormone (LH) binds to its receptors in theca cells to produce testosterone, while follicle stimulating hormone (FSH) binds to its receptors in granulosa cells to promote conversion of testosterone to estradiol [15].

Nanoparticle accumulation and toxicity in the ovary has been documented to occur in recent studies [16]. Interestingly, one study found that NPs can reach specific localizations within the ovary. Specifically, the authors found NPs accumulating in round, ball-like structures with a diameter of approximately 200–300 µm, suggesting their accumulation in growing follicles such as antral follicles [17]. ZnO NPs have been found to be internalized in cultured ovarian cells after 24 h of exposure [18]. Another study found ZnO NP internalization in hen ovarian tissue after in vivo exposure to 1–200 mg/kg body weight [19]. Previous in vitro studies have shown that ZnO NP (37 nm, range 13–89 nm) exposure temporarily decreases metabolic activity and follicular growth, affects cytoskeletal arrangement, and induces ultrastructural alterations in antral follicles [20]. However, whether exposure to these novel materials disrupts antral follicle functions is unknown. Thus, the objective of this study was to test the hypothesis that in vitro exposure to ZnO NPs affects the steroidogenic pathway and induces oxidative stress in ovarian antral follicles. In addition, ZnO NP physicochemical characteristics may influence NP toxicity as they might dissolve in culture media before and/or after internalization. Thus, we determined NP dissolution over time in culture.

## 2. Materials and Methods

### 2.1. Chemicals

Insulin transferrin selenium (ITS), penicillin/streptomycin (P/S), phosphate-buffered saline (PBS) and paraformaldehyde were purchased from Sigma-Aldrich (St. Louis, MO, USA). Alpha minimal essential medium (α-MEM) was obtained from Thermofisher (Waltham, MA, USA). Human recombinant follicle stimulating hormone (rFSH) was obtained from Dr. A.F. Parlow from the National Hormone and Peptide Program (Harbor-UCLA Medical Center, Torrance, CA, USA). Fetal bovine serum (FBS) was obtained from Atlanta Biologicals (Lawrenceville, GA, USA).

### 2.2. Nanoparticle Suspension and Physicochemical Characterization

ZnO NPs used in this study were a kind gift from Dr. Lutz Mädler (University of Bremen Germany), and they were synthesized by flame spray pyrolysis. A full characterization of ZnO NPs was previously performed [20]. Briefly, ZnO NPs elemental composition and crystalline structure were confirmed by energy dispersive spectroscopy (EDS) in a transmission electron microscope and by X-ray diffraction analyses. ZnO NPs showed a primary size of 37 nm, with a size distribution ranging between 13 and 89 nm, confirming their nanometric size. ZnO NPs exhibit spheroidal or irregular forms in a hexagonal zincite crystalline structure. A sonication protocol was established to avoid NP agglomeration, and suspension stability in culture media was assessed [20]. ZnO NP suspension in the culture media showed a hydrodynamic diameter (HD) of 133 nm at the lowest concentration (5 µg/mL), and a HD of 73 nm at the highest concentration (15 µg/mL).

### 2.3. Animals

Adult CD-1 female mice (28–30 days old) were purchased from Charles River Laboratories (Wilmington, MA, USA). Mice were housed in polysulfonate cages and maintained under 12 h dark-light cycles, a temperature of 22 ± 1 °C and 50% of relative humidity at the University of Illinois Urbana-Champaign, College of Veterinary Medicine Animal Facility. Food and high purity water were provided ad libitum. All animal procedures were approved by the Institutional Animal Care and Use Committee at the University of Illinois Urbana-Champaign in compliance with international guidelines for the use and care of laboratory animals.

### 2.4. Antral Follicle Culture

Female mice (two per independent experiment) were euthanized by CO_2_ and then their ovaries were aseptically removed. Antral follicles (220–400 µm) were mechanically isolated from ovaries in culture media and cleaned of interstitial tissue using fine watchmaker forceps. Antral follicles (15–20 per ovary) were pooled and individually placed in 96-well culture plates (Corning Inc.) to complete at least 12 follicles per treatment group in at least three independent experiments. Antral follicles were cultured as previously described [20], with minor modifications. Briefly, antral follicles were cultured in an incubator with 5% CO_2_ at 37 °C. On culture day 0, all follicles, independently of the treatment group, were cultured in α-MEM supplemented with ITS (insulin:10 ng/mL, transferrin: 10 ng/mL, selenium: 10 pg/mL), P/S (penicillin: 100 U/mL, streptomycin: 100 µg/mL), 5% FBS and rFSH (5 IU/mL). On culture day 3, media were removed and substituted with supplemented media plus ZnO NPs at different concentrations (5, 10, and 15 µg/mL). Antral follicle cultures were maintained for 4 more days (96 h of NP exposure) until day 7 of culture. At the end of the culture (96 h of exposure), media were collected and stored at −80 °C until subjected to sex steroid hormone assays. Antral follicles were pooled at collection, snap frozen, and stored at −80 °C until gene expression analyses.

### 2.5. Analyses of Sex Steroid Hormone Levels

Culture media collected at 96 h were subjected to immunosorbent assays (ELISAs, DRG International Inc., Springfield, NJ, USA). Analytical sensitivities for progesterone, androstenedione, testosterone, and estradiol were 0.045, 0.019, 0.083, and 0.0097 ng/mL, respectively. Both intra- and inter-assay coefficients of variation were below 10%, except for androstenedione analyses which were below 15%. Samples were run in duplicate and diluted if needed to fit the dynamic range of the ELISA kits.

### 2.6. Analyses of Gene Expression by Quantitative Polymerase Chain Reaction (qPCR)

Total RNA was isolated from antral follicles (12–15 follicles per treatment from at least 3 independent experiments) using a RNeasy Micro kit (Qiagen, Inc., Valencia, CA, USA) following the manufacturer’s instructions. RNA was eluted using 14 µL of RNase-free water, and the concentration was determined using a nanodrop (λ = 260/280 nm; ND one; Nanodrop Technologies Inc., Wilmington, DE, USA). Total RNA (100 ng) was reversed transcribed to complementary DNA (cDNA) using the iScript RT kit (Bio-Rad Laboratories, Inc., Hercules CA, USA) according to manufacturer’s protocol. Analysis of qPCR was conducted using the C and FX96 Real-Time PCR Detection System (Bio-Rad Laboratories, Inc, Hercules CA, USA) and accompanying CFX Manager Software according to the manufacturer’s protocol. The equipment quantifies the amount of PCR product by measuring SsoFastEvaGreen dye (Bio-Rad Laboratories) that fluoresces when bound to double-stranded DNA. All qPCR reactions were conducted in duplicate, in a 10 µL reaction volume containing 1.67 ng of cDNA, forward and reverse primers (0.75 pmol) for all genes specified in Table 1, and SsoFastEvaGreen Supermix. The qPCR program consisted of an enzyme activation step (95 °C for 5 min), an amplification and quantification program (39 cycles of 94 °C for 10 s, 60 °C for 10 s, and 72 °C for 10 s), a melt curve (65 °C to 95 °C heating, 0.5 °C/s with continuous fluorescence readings), and a final step at 72 °C for 5 min per the manufacturer’s protocol. All gene expression data were normalized to the housekeeping gene (actin, beta). Relative fold changes were calculated as the ratio of each treatment group to the control group level from the same culture and were analyzed using a mathematical model for relative quantification of qPCR data developed by Pfaffl [21].

### 2.7. Analyses of Reactive Oxygen Species Levels (ROS)

Antral follicle ROS levels were measured using a fluorometric kit (Cell Biolabs STA-347, San Diego, CA, USA) according to manufacturer’s instructions using at least 30 antral follicles per treatment group from three independent experiments. ROS/reactive nitrogen species (RNS) from samples react with dichlorodihydrofluorescein (DCFH), which is rapidly oxidized to the highly fluorescent 2′7′-dicholorodihydrofluoresceine (DCF). Fluorescence intensity is proportional to the total ROS/RNS species within samples. Briefly, after 96 h of exposure, antral follicles were washed with PBS to remove NPs. Then, follicles were pooled, homogenized in PBS, and centrifuged to remove insoluble particles. Homogenates were added to a 96 well plate in duplicate using standards, positive (H_2_O_2_ 20 µM), negative (PBS), and NP (ZnO NPs 15 µg/mL) controls. After a brief incubation, DCFH probe was added to all wells, and the oxidation reaction was allowed to proceed. Samples were measured fluorometrically against a DCF standard curve. Data were normalized to protein concentrations per well in the ROS measuring assay and presented as relative to the control. Protein concentrations were quantified using the BCA protein assay.

### 2.8. Inductively Coupled Plasma Mass Spectrometry (ICP-MS) Analyses

To determine ZnO NP dissolution in the culture media, suspensions containing 5 and 15 µg/mL ZnO NPs were incubated in culture media for 1, 24, 48, and 96 h. Thereafter, media were collected and centrifuged at 15,000× *g* for 1 h to precipitate NPs. The supernatant was used to quantify Zn as an indicator of its dissolution from ZnO NPs. To estimate Zn internalization by follicles from ZnO NP exposure, cultured antral follicles were washed three times with PBS to remove any NPs from the follicle surface (not internalized). Antral follicles and collected media were pooled by treatment group and stored at −70 °C until analysis. For ICP-MS analysis, samples were diluted (1:10 *v*/*v*) using ultrapure water and 65% concentrated nitric acid (final concentration of 0.16%) (Merck, Darmstadt, Hesse, Germany). Then, the samples were analyzed using an ICP-MS NexION 300D (Perkin Elmer, Waltham, MA, USA) in a certified laboratory of metals (Accreditation Number: INV-0007-013/19, based in NMX-EC-17025-IMNC-2018 standard—ISO/IEC 17025:2017), at the Laboratory of Research and Services in Toxicology (LISTO), Cinvestav. Certified reference materials (QM-U-Q1904, QM-U-Q1905, QM-U-Q1906) from the National Public Health Institute of Quebec were used as quality control. All measurements, including those of the samples, standards and reference materials, were performed in duplicate and analyzed using the Syngistix software (Perkin Elmer, Waltham, MA, USA).

### 2.9. Statistical Analyses

All data analyses were performed using SPSS statistical software (SPSS Inc., Chicago, IL, USA) and/or GraphPad Prism 9.4.1 software (GraphPad Software, Inc., San Diego, CA, USA). Data were presented as the mean ± SEM (standard error of the mean) from three independent experiments with at least 12 follicles per group. Since data were normally distributed, multiple comparisons between experimental groups were made using one-way analysis of variance (ANOVA) followed by Dunnett post hoc comparisons. Statistical significance was assigned at *p* ≤ 0.05. When comparisons between two groups were performed, a Student *t*-test was used.

## 3. Results

### 3.1. Effect of Nanoparticles on Antral Follicle Apoptosis and Proliferation

**Figure 1:**
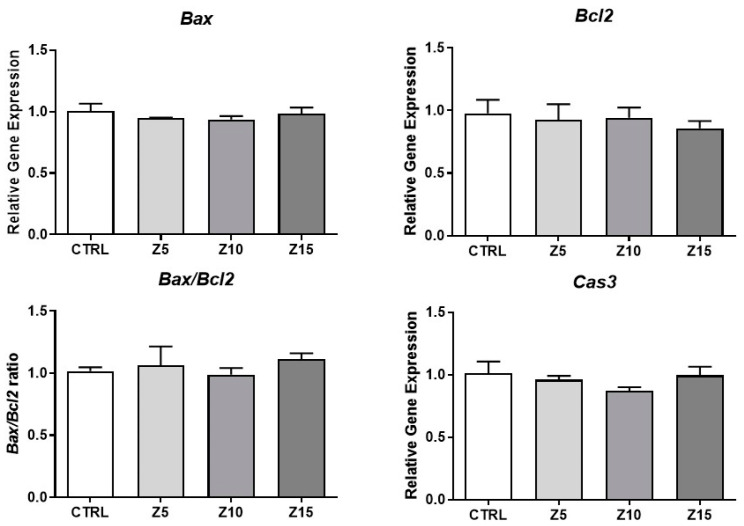
Effects of ZnO NP exposure on apoptosis regulators in antral follicles. Antral follicles were cultured in vitro and exposed to ZnO NPs at 5, 10, and 15 µg/mL. RNA was extracted from follicles, reversed transcribed to cDNA, and subjected to qPCR. mRNA levels of *Bax*, *Bcl2*, and *Cas3* were assessed. Graphs represent means ± SEM from three independent experiments (12–15 follicles per group). No significant differences were shown after ANOVA comparisons. CTRL: control, Z5: ZnO NPs 5 µg/mL, Z10: ZnO NPs 10 µg/mL, Z15: ZnO NPs 15 µg/mL.

**Figure 2:**
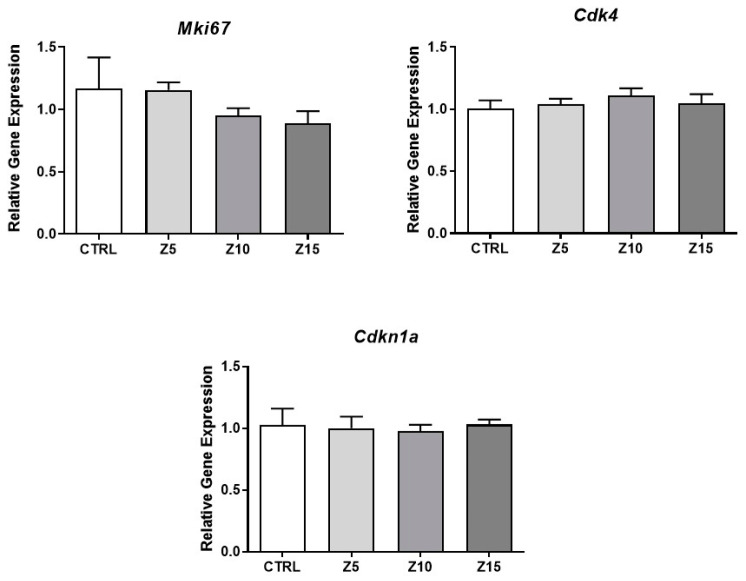
Effects of ZnO NP exposure on cell cycle regulators in antral follicles. Antral follicles were cultured in vitro and exposed to ZnO NPs at 5, 10, and 15 µg/mL. RNA was extracted from follicles, reversed transcribed to cDNA, and subjected to qPCR. mRNA levels of *Ki67*, *Cdk4*, and *Cdkn1a* were assessed. Graphs represent means ± SEM from three independent experiments (12–15 follicles per group). No significant differences were shown after ANOVA comparisons. CTRL: control, Z5: ZnO NPs 5 µg/mL, Z10: ZnO NPs 10 µg/mL, Z15: ZnO NPs 15 µg/mL.

Antral follicle growth is dependent on cell proliferation and apoptosis. Alterations in apoptosis and/or the cell cycle are related to changes in follicular diameter and steroidogenic capacity [22]. Previous studies have shown that ZnO NP exposure induces apoptosis [23]. In addition, we previously found that ZnO NP exposure induces a temporary decrease in antral follicle growth [20]. Thus, we evaluated apoptotic markers and cell cycle regulators to determine whether ZnO NPs induce apoptosis and/or cell proliferation alterations in antral follicles. None of the tested ZnO NP concentrations (5, 10, and 15 µg/mL) induced apoptosis in antral follicles compared to control, as evidenced by no changes in *Bax*, *Bcl2*, *Bax*/*Bcl2* ratio, and *Cas3* mRNA levels (Figure 1). None of the tested ZnO NP concentrations affected cell proliferation, as evidenced by similar *Mki67*, *Cdk4*, and *Cdkn1a* mRNA levels compared to control (Figure 2).

### 3.2. Effect of Nanoparticles on Sex Steroid Hormone Levels

**Figure 3:**
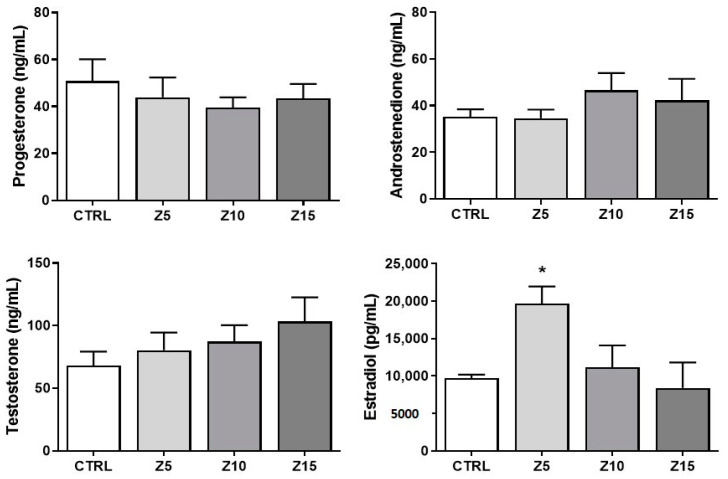
Effects of ZnO NP exposure on sex steroid hormone levels. Culture media were subjected to enzyme-linked immunosorbent assays. Graphs represent means ± SEM from three independent experiments (12–15 follicles per group). The asterisk (*) indicates significant difference from the control (*p* ≤ 0.05). CTRL: control, Z5: ZnO NPs 5 µg/mL, Z10: ZnO NPs 10 µg/mL, Z15: ZnO NPs 15 µg/mL.

We evaluated sex steroid hormone levels to determine potential alterations in follicular sex steroid synthesis induced by ZnO NP exposure (Figure 3). None of the tested ZnO NP concentrations altered thecal-cell-derived hormones such as progesterone, androstenedione, and testosterone. Interestingly, the lowest concentration of ZnO NPs (5 µg/mL) altered the granulosa-cell derived hormone estradiol by increasing its levels in culture media collected from exposed follicles compared to that of controls (Figure 3). None of the higher concentrations of ZnO NPs affected estradiol levels compared to control (Figure 3).

### 3.3. Effect of Nanoparticles on Steroidogenic Involved Genes

**Figure 4:**
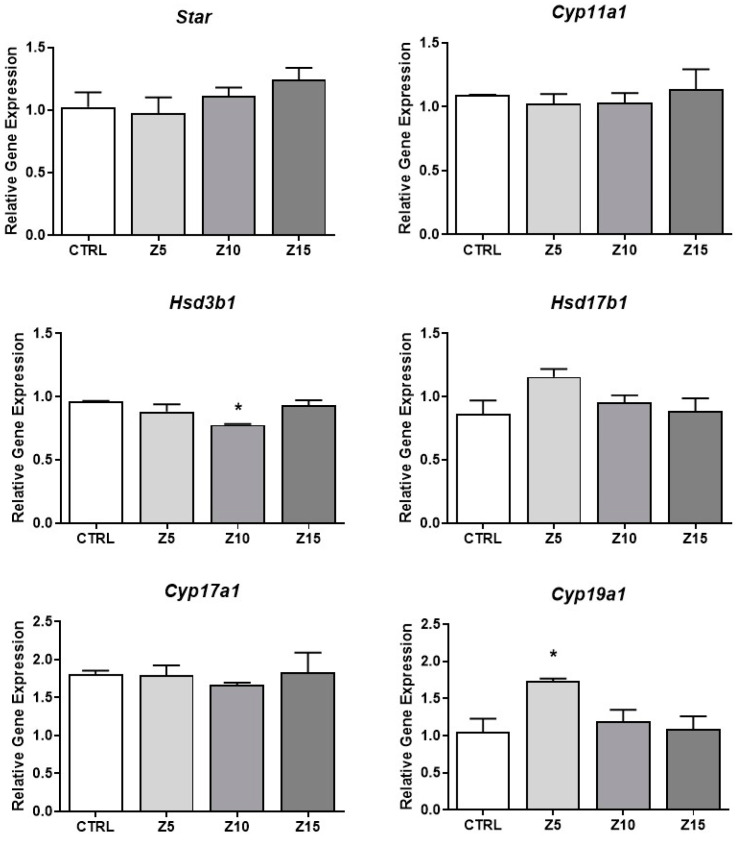
Effects of ZnO NP exposure on steroidogenesis regulators in antral follicles. Antral follicles were cultured in vitro and exposed to ZnO NPs at 5, 10, and 15 µg/mL. RNA was extracted from follicles, reversed transcribed to cDNA, and subjected to qPCR. mRNA levels of *Star*, *Cyp11a1*, *Hsd3b1*, *Hsd17b1*, *Cyp17a1*, and *Cyp19a1* were assessed. Graphs represent the mean ± SEM from three independent experiments (12–15 follicles per group). Asterisks (*) indicate significant differences compared to control (*p* ≤ 0.05). CTRL: control, Z5: ZnO NPs 5 µg/mL, Z10: ZnO NPs 10 µg/mL, Z15: ZnO NPs 15 µg/mL.

To investigate the mechanism by which ZnO NPs increase estradiol levels, we evaluated expression of the enzymes involved in the estradiol synthesis pathway. We found that none of the tested ZnO NP concentrations affected mRNA levels of *Star*, *Cyp11a1*, *Hsd17b1*, and *Cyp17a1* compared to control (Figure 4). However, ZnO NPs at 10 µg/mL decreased expression of *Hsd3b1* compared to control. Further, ZnO NPs at the lowest concentration (5 µg/mL) significantly increased the expression of *Cyp19a1* compared to control (Figure 4).

### 3.4. Effect of ZnO Nanoparticles on Hormone Receptor mRNA Levels

**Figure 5:**
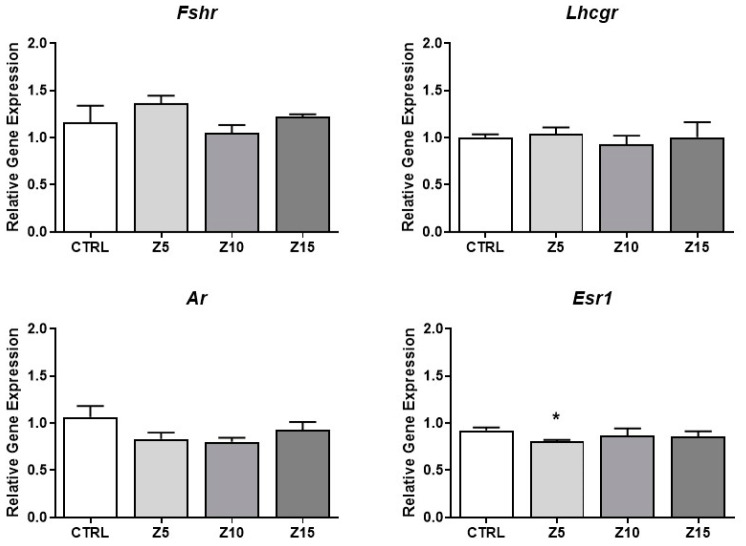
Effects of ZnO NP exposure on hormone receptors in antral follicles. Antral follicles were cultured in vitro and exposed to ZnO NPs at 5, 10, and 15 µg/mL. RNA was extracted from follicles, reversed transcribed to cDNA, and subjected to qPCR. mRNA levels of *Fshr*, *Lhcgr*, *Ar*, and *Esr1* were assessed. Graphs represent mean ± SEM from three independent experiments (12–15 follicles per group). Asterisks (*) indicate significant differences compared to control (*p* ≤ 0.05). CTRL: control, Z5: ZnO NPs 5 µg/mL, Z10: ZnO NPs 10 µg/mL, Z15: ZnO NPs 15 µg/mL.

Hormone synthesis is tightly regulated at different levels, including the levels of hormone receptors [24]. Thus, we evaluated the effects of ZnO NPs on the expression of key steroid hormone receptors in antral follicles. None of the tested concentrations of ZnO NPs affected the expression of the *Fshr*, *Lhcgr*, and *Ar*. However, the lowest concentration (5 µg/mL) of ZnO NPs decreased *Esr1* expression compared to the control group (Figure 5).

### 3.5. Effect of Nanoparticles on Antioxidant Enzymes Expression Levels

**Figure 6:**
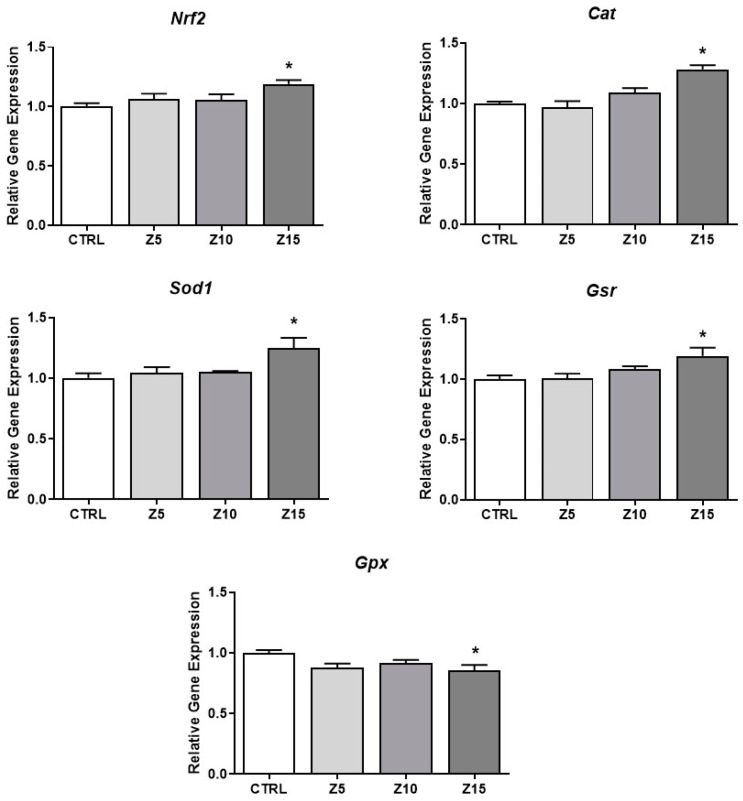
Effects of ZnO NP exposure on antioxidant proteins in antral follicles. Antral follicles were cultured in vitro and exposed to ZnO NPs at 5, 10, and 15 µg/mL. RNA was extracted from follicles, reversed transcribed to cDNA, and subjected to qPCR. mRNA levels of *Nrf2*, *Cat*, *Sod1*, *Gsr*, and *Gpx* were assessed. Graphs represent means ± SEM from three independent experiments (12–15 follicles per group). Asterisks (*) indicate significant differences compared to control (*p* ≤ 0.05). CTRL: control, Z5: ZnO NPs 5 µg/mL, Z10: ZnO NPs 10 µg/mL, Z15: ZnO NPs 15 µg/mL.

ZnO NPs have been shown to induce oxidative stress [25]. To determine if ZnO NP exposure at the tested concentrations induced oxidative stress in the antral follicles, we evaluated antioxidant responses. The highest concentration of ZnO NPs (15 µg/mL) induced the expression of *Nrf2*, an important nuclear factor involved in regulation of cellular resistance to oxidants [26], induced the expression of the antioxidant enzymes *Cat*, *Sod1*, and *Gsr*, and decreased the expression of the antioxidant enzyme *Gpx* compared to the control (Figure 6).

### 3.6. Effect of Nanoparticles on ROS Levels

**Figure 7:**
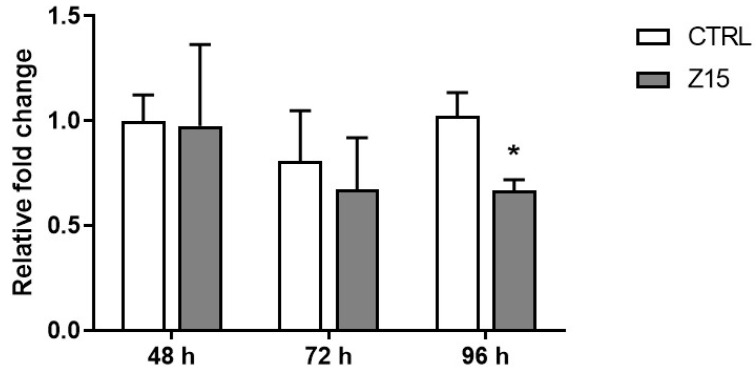
Effects of ZnO NP exposure on reactive oxygen species in antral follicles. Antral follicles were cultured in vitro and exposed to 15 µg/mL ZnO NPs for 48, 72, and 96 h. Antral follicles were collected, and processed for ROS/RNS levels. Data were normalized to protein concentration and presented as relative to control. Graph represents means ± SEM from three independent experiments (12–15 follicles per group). Asterisks (*) indicate significant differences compared to control for the same exposure time (*p* ≤ 0.05).

Based on the above results, we next determined if exposure to ZnO NPs at 15 µg/mL induced the formation of ROS in cultured antral follicles at different timepoints. Exposure to ZnO NPs (15 µg/mL) did not induce ROS after 48 h and 72 h. However, exposure to ZnO NPs (15 µg/mL) for 96 h resulted in a significant decrease in the relative levels of reactive oxygen/nitrogen species compared to control (Figure 7).

### 3.7. ZnO Nanoparticle Dissolution in Culture Media

**Figure 8:**
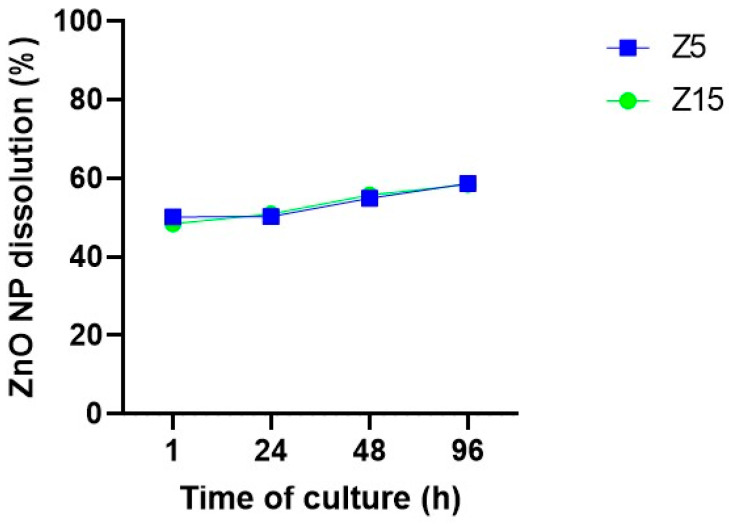
Zinc dissolution from ZnO NPs in culture media over time. ZnO NPs at 5 and 15 µg/mL were incubated in culture media for 1, 24, 48, and 96 h. Then, the supernatant resulting from centrifugation was subjected to ICP-MS to determine Zn ions levels. Data are presented as percentage of the original concentration added to the culture media.

Physicochemical characteristics such as dissolution and aggregation effects are relevant for toxicological outcomes of NPs [27,28]. Based on previous observations [20], ZnO NP dissolution was assessed in our system to determine whether NPs are being internalized as NPs and/or as ions. Zinc levels on centrifuged media were evaluated after 1, 24, 48, and 96 h of culture to determine the time course of ZnO NP dissolution. Our data show that ZnO NP dissolution starts early in the culture, with 48% and 50% dissolution after 1 h of culture for 15 and 5 µg/mL, respectively. NP dissolution increased up to ~58% after 96 h of culture (Figure 8).

### 3.8. ZnO NP Internalization on Antral Follicles

**Figure 9:**
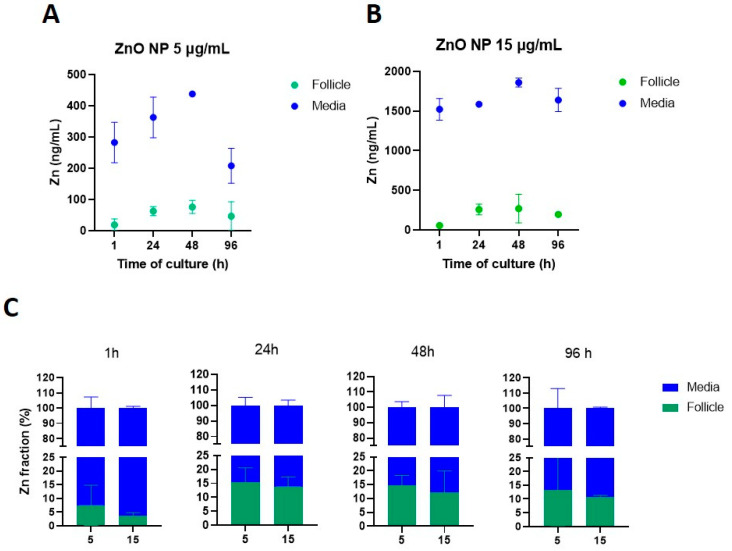
Zinc (Zn) internalization from ZnO NPs into cultured antral follicles. Antral follicles were exposed to ZnO NPs (5 and 15 µg/mL) for 1, 24, 48, and 96 h. Following follicle washing protocols to collect the supernatant from culture media, the corresponding samples were subjected to ICP-MS to quantify Zn levels. Zinc levels are presented as concentrations in antral follicles and culture media (panels (**A**) and (**B**), respectively), and as fractions of the total Zn content in the system over time (**C**) from incubations with 5 and 15 µg/mL ZnO NPs. Graphs represent means ± SEM from two independent experiments (12–15 follicles per group).

We then evaluated the fractions of Zn in the culture media and the follicular cells. Before exposure to ZnO NPs, Zn concentrations in either culture media or antral follicles were below the detection limit value (LD = 0.368 ng/mL). For the lowest ZnO NP concentration (5 µg/mL), Zn mean concentrations in culture media ranged between 208 and 437 ng/mL, whereas Zn mean concentration in antral follicles ranged between 19 and 75 ng/mL (Figure 9A). At the highest concentration (15 µg/mL), Zn mean concentrations in culture media ranged between 1523 and 1858 ng/mL, whereas those in the antral follicles were 55–269 ng/mL (Figure 9B).

We also evaluated the fraction of Zn in the culture media and the fraction internalized by antral follicles (Figure 9C). After 1 h of exposure, the lowest ZnO NP concentration showed higher levels of internalization to the antral follicles (7.4%) compared to the highest ZnO NP concentration (3.62%). However, internalization was similar between the two ZnO NP exposed groups after 24 h (15.3 and 13.8%, for ZnO NPs 5 and 15 µg/mL, respectively), 48 h (14.6% and 12.2% for ZnO NPs 5 and 15 µg/mL, respectively), and 96 h (13.1% and 10.6% for ZnO NPs 5 and 15 µg/mL, respectively) (Figure 9C). Although Zn concentrations were higher in antral follicles from the highest concentration (Figure 9B), the internalized fraction was higher in the lowest concentration (Figure 9C).

## 4. Discussion

This study builds on previous findings [20] and investigates the effects on the antral follicle steroidogenic capacity through the mechanism of oxidative stress. Specifically, our previous study showed that ZnO NP exposure results in morphological alterations in antral follicles and suggested that NPs caused oxidative stress induction. In addition, we observed an apparent dissolution of ZnO NPs in the culture media [20]. Given that the physicochemical characteristics of NPs are known to influence their toxic effects [29], we delved deeper into this observation. Thus, we conducted a detailed investigation of ZnO NPs dissolution over time in antral follicle culture conditions.

Studies have suggested extracellular dissolution as responsible for the ZnO NP-mediated cytotoxicity [25,30]. Contrastingly, other studies have demonstrated continued ZnO NP uptake and subsequent dissolution in the acidic cell compartments of cells, causing disruption of the cytosolic Zn levels [31]. In our study, we evaluated the dissolution of ZnO NPs over time and found that approximately 50% dissolved as soon as 1 h of NP suspension and throughout the culture. This finding suggests that ZnO NPs dissolve to favor Zn^2+^ internalization into the ovarian follicle as a potential mechanism of toxicity. Although previous analyses of exposed antral follicles via electron microscopy did not show clear evidence of ZnO NPs present inside the cells, they showed damage at the ultrastructure level (i.e., the zona pellucida) [20]. Studies suggest that NP dissolution in media often results in a complex suspension. This suspension can contain free and complex ions derived from the NPs, partially dissolved NPs, and ions adsorbed on the NP surface [28]. Therefore, while our focus is on dissolution, we cannot disregard potential effects due to NP internalization. Based on the above, ZnO NPs in our testing system led to a complex mixture of NPs and Zn^2+^ to induce antral follicle toxicity.

Different studies have shown that NPs can induce oxidative stress in different cells and tissues [10,32,33]. To determine whether ZnO NP exposure results in oxidative stress in antral follicles, we performed direct and indirect evaluations. We evaluated the antral follicle antioxidant response by determining the mRNA levels of antioxidant enzymes. In addition, we directly evaluated the reactive oxygen species levels at different timepoints of culture. After 96 h of ZnO NP exposure, the highest concentration (15 µg/mL) showed effects on the antioxidant enzymes, as evidenced by increased mRNA levels on *Nrf2*, *Cat*, *Sod1*, and *Gsr*. Those data were further supported by the decreased mRNA levels of *Gpx*, and decreased ROS/RNA levels. Zinc, as NPs and as ions, can affect the oxidative balance response. Engineered NPs possess a small size, high surface area, and high surface reactivity, leading to the production of ROS [10]. In a study performed on a primary culture of rat astrocytes exposed to Zn acetate, the inactivation of the enzyme Gsr, an increased GSSH:GSH ratio, and increased ROS levels were observed at concentrations higher than 150 µM of Zn [34]. In another study, ZnO NP exposure (25 and 50 µg/mL) from 1 to 24 h decreased mRNA levels of antioxidant enzymes (*Sod2*, *Cat*, *Gpx*) in SHSY5Y cells [32]. In another study using fish, ROS generation was reported after 10-day exposure to ZnO NPs (300–1000 mg/kg) [33]. The above studies gather evidence of Zn, as NPs and as ions, altering the oxidative balance in different biological systems. In addition, our data suggest that after 96 h of ZnO NP exposure at the highest concentration, an antioxidant response has been induced, resulting in decreased ROS levels. The above effects on oxidative status could be related, at least in part, to the observed release of Zn ions into the media. Zinc is a divalent ion that does not directly participate in oxidation-reduction reactions [35]. However, Zn particles can regulate iron homeostasis due to a tight relationship between factors regulating both metals [36]. Additionally, different studies have shown that exposure to different NPs can interfere with essential metals homeostasis [37,38]. It was reported that both Zn ions and ZnO NPs can contribute to ZnO NP toxicity in HUVEC cells by affecting iron homeostasis and inducing ferroptosis through elevation of ROS [39]. It is possible that Zn both as an ion or as a NP is affecting iron homeostasis, promoting oxidation reactions, and oxidative stress induction, leading to the observed antioxidant enzyme response.

When ZnO NP effects on antral follicle steroidogenesis were evaluated, we found a clear and well-defined effect at the lowest tested ZnO NP concentration. Exposure to 5 µg/mL ZnO NPs increased estradiol levels due to induction of *Cyp19a1*. The other tested ZnO NP concentrations did not affect hormone levels. ZnO NPs may be affecting estradiol levels as NPs and/or as free Zn. In a study using rat granulosa cells exposed to gold NPs, the authors found increased levels of estradiol compared to control after 5 h of exposure, suggesting direct NP interaction with mitochondria and lipid droplets, leading to altered permeability and increased estradiol concentrations [40]. Zinc has been shown to regulate genes participating in steroidogenesis. The role of Zn in regulating androgen metabolism and aromatization has been shown [41,42,43]. One study found decreased estradiol and LH serum levels, as well as decreased androgen receptors and increased estrogen receptors in livers from rats with a Zn deficient diet [41]. Another study using zebra fish embryos as models found that Zn supplement induces the expression of *Cyp19a1* after cadmium inhibitory effects [42]. Recently, another study described Zn participation on its own transporters (ZnT7) in Leydig cells and found that Zn levels regulate the expression of genes such as *Cyp11a1* and *Hsd3b1*; thus, affecting testosterone levels [43]. Here, we show that ZnO NP exposure resulted in modulation of the steroidogenic pathway at the transcriptional level. Based on the above, knowing that up to 50% of ZnO NPs are dissolving, and that no NPs were found internalized through electron microscopy analysis [20], it seems plausible that ZnO NPs are modulating estradiol synthesis mainly as free Zn at the lowest tested concentration. However, because ZnO NPs do not fully dissolve in our system, and because detrimental effects caused by ZnO NPs have been described compared to free Zn ions, we cannot rule out any influence of the ZnO as NP on the effects observed in this study.

Interestingly, we also found that the ZnO NP concentration (5 µg/mL) that increased estradiol levels also decreased *Esr1* mRNA levels. In a study, the effect of estradiol on *Esr1* expression in uterine tissue was evaluated and found that ovariectomized immature rats treated with estradiol had different outcomes over time. After 3 h, there was a decrease in *Esr1* mRNA levels, while there was an increase after 24 h with a posterior decrease after 72 h on Esr1 mRNA levels [44], showing the differential regulation of estradiol levels on *Esr1* mRNA levels over time. Together, exposure to ZnO NPs at the lowest concentration for 96 h could result in Zn induction of estradiol, leading to a negative regulation on *Esr1* mRNA levels. Another study using rats found that exposure to ZnO NPs increased serum estradiol levels at low doses (4 and 8 mg/kg BW), whereas it decreased estradiol levels at higher concentrations (100 and 200 mg/kg BW) [45], showing a nonmonotonic response. Endocrine disruptors have nonmonotonic dose response [7]. NPs are increasingly known to have potential as endocrine disruptor chemicals [7]. Thus, the above could explain our data showing no effects at the highest tested ZnO NP concentrations. The effects on *Esr1* mRNA levels at the lowest concentration and the lack of effects at the higher ones could result from the regulation of the mRNA molecule stability. One study exposing MCF-7 cells to estradiol (10^−9^ M) for 6 h resulted in decreased *Esr* mRNA half-life from 4 h in control to 40 min in the treated cells [46].

In our study, we assessed mRNA levels of genes coding for apoptotic (*Bcl2*, *Bax* or *Cas3*) and cell cycle regulators (*Cdk4*, *Cdkn1*, and *Mki67*), but the levels were similar to their control groups. Apoptosis and cell cycle alterations have been described after exposure to different NPs. One study using human ovarian cells (SKOV3) exposed to ZnO NPs showed induced apoptotic markers and reduced cell viability in a dose-dependent manner [23]. Another study revised that exposure to most used metallic NPs, including silver-, gold-, TiO_2_-, and ZnO-NPs, is directly associated with cell cycle alterations and DNA damage linked to cell cycle arrest and cell death [47].

It is worth noting that the antral follicle culture is a primary culture and a well-recognized model retaining steroidogenic activity for several days. This in vitro system allows for a better understanding of the interaction of ZnO NPs with antral follicles. Thus, its use aids us to understand the fundamental biological functions of the antral follicles and the toxic effects of toxic compounds. Future studies should address the ovarian cell outcomes of ZnO NP exposure in a whole complex in vivo system.

## 5. Conclusions

In conclusion, we found that ZnO NPs dissolve and may internalize into antral follicles, leading to increased estrogen production and an antioxidant response. Dissolution in culture media and follicle internalization were found as soon as 1 h exposure. Exposure to the lowest concentration of ZnO NPs increased levels of estradiol and mRNA levels of *Cyp19a1*. Collectively, these data emphasize the importance of evaluating several concentrations of NPs since NP physicochemical characteristics might result in toxic outcomes.

## Figures and Tables

**Table 1:** Genes and primer sequences used in qPCR.

Gene Name	Symbol	Forward Primer	Reverse Primer
Actin, beta	*Actb*	5′-GGGCACAGTGTGGGTGAC-3′	5′-CTGGCACCACACCTTCTAC-3′
Steroidogenic acute regulatory protein	*Star*	5′-CAGGGAGAGGTGGCTATGCA-3′	5′-CCGTGTCTTTTCCAATCCTCTG-3′
Cytochrome-P450 cholesterol side-chain cleavage	*Cyp11a1*	5′-AGATCCCTTCCCCTGGTGACAATG-3′	5′-CGCATGAGAAGAGTATCGACGCATC-3′
Cytochrome P450 steroid 17-α-hydroxylase 1	*Cyp17a1*	5′-CCAGGACCCAAGTGTGTTCT-3′	5′-CCTGATACGAAGCACTTCTCG-3′
3β-hydroxysteroid dehydrogenase 1	*Hsd3b1*	5′-CAGGAGAAAGAACTGCAGGAGGTC-3′	5′-GCACACTTGCTTGAACACAGGC-3′
17β-hydroxysteroid dehydrogenase 1	*Hsd17b1*	5′-AAGCGGTTCGTGGAGAAGTAG-3′	5′-ACTGTGCCAGCAAGTTTGCG-3′
Cytochrome P450 aromatase	*Cyp19a1*	5′-CATGGTCCCGGAAACTGTGA-3′	5′-GTAGTAGTTGCAGGCACTTC-3′
Nuclear respiratory factor 1	*Nrf2*	5′-TGAAGCTCAGCTCGCATTGA-3′	5′-TGCTCCAGCTCGACAATGTT-3′
Catalase	*Cat*	5′-GCAGATACCTGTGAACTGTC-3′	5′-GTAGAATGTCCGCACCTGAG-3′
Glutathione reductase	*Gsr*	5′-CAGTTGGCATGTCATCAAGCA-3′	5′-CGAATGTTGCATAGCCGTGG-3′
Superoxide dismutase 1	*Sod1*	5′-TTCCGTCCGTCGGCTTCTCGT-3′	5′-CGCACACCGCTTTCATCGCC-3′
Glutathione peroxidase	*Gpx*	5′-CCTCAAGTACGTCCGACCTG	5′-CAATGTCGTTGCGGCACACC-3′
Bcl2-associated X protein	*Bax*	5′-TGAAGACAGGGGCCTTTTTG	5′-AATTCGCCGGAGACACTCG-3′
B cell leukemia/lymphoma 2	*Bcl2*	5′-ATGCCTTTGTGGAACTATATGGC	5′-GGTATGCACCCAGAGTGATGC-3′
Caspase 3	*Cas3*	5′-TGGTGATGAAGGGGTCATTTATG	5′-TTCGGCTTTCCAGTCAGACTC-3′
Marker of proliferation Ki-67	*Mki67*	5′-GCTCACCTGGTCACCATCAA	5′-ACTACAGGCAGCTGGATACG-3′
Cyclin-dependent kinase 4	*Cdk4*	5′-AGAAACCCTCGCTGAAGCGGCA	5′-TGGGGGTGAACCTCGTAAGGAGA-3′
Cyclin-dependent kinase inhibitor 1A (P21)	*Cdkn1a*	5′-TTAGGCAGCTCCAGTGGCAACC	5′-ACCCCCACCACCACACACCATA-3′
FSH receptor	*Fshr*	5′-AGCAAGTTTGGCTGTTATGAGG	5′-GTTCTGGACTGAATGATTTAGAGG-3′
LH receptor	*Lhcgr*	5′-AACCCGGTGCTTTTTACAAACC	5′-TCCCATTGAATGCATGGCTT-3′
Androgen receptor	*Ar*	5′-GGCGGTCCTTCACTAATGTCAACT	5′-GAGACTTGTGCATGCGGTACTCAT-3′
Estrogen receptor 1 (alpha)	*Esr1*	5′-AATTCTGACAATCGACGCCAG	5′-GTGCTTCAACATTCTCCCTCCTC-3′

## Data Availability

Not applicable.
